# Preparation of MWCNT/CoMn_2_O_4_ nanocomposite for effectual degradation of picric acid via peroxymonosulfate activation

**DOI:** 10.1038/s41598-024-62351-1

**Published:** 2024-05-20

**Authors:** Ayda Farajollahi, Ahmad Poursattar Marjani

**Affiliations:** Department of Organic Chemistry, Faculty of Chemistry, Urmia University, Urmia, Iran

**Keywords:** MWCNT/CoMn_2_O_4_, Dye removal, Picric acid, Proxymonosulfate (PMS), Advanced oxidation processes (AOPs), Environmental chemistry, Green chemistry

## Abstract

In recent years, using nanomaterials based on multi-wall carbon nanotubes (MWCNT) through the activation of peroxymonosulfate (PMS) has attracted more attention to the degradation of organic pollutants. This research presented a new route for the synthesis of MWCNT/CoMn_2_O_4_ nanocomposite for the degradation of picric acid using advanced oxidation processes (AOPs). Firstly, CoMn_2_O_4_ nanoparticles were prepared and then loaded on MWCNT using ultrasonic waves. The results of various analyzes confirmed the successful loading of nanoparticles on carbon nanotubes. As the degradation process proceeds through oxidation processes, the high electronic conductivity of MWCNT and the active sites of Mn and Co in the nanocomposite play an essential role in activating PMS to generate reactive oxygen species (ROS). An investigation of the reaction mechanism in different conditions showed that the highest speed of picric acid decomposition in the presence of nanocomposite (98%) was in 47 min. However, the scavenger test showed that HO^·^ and SO_4_^·−^ radicals are more important in the degradation process. Meanwhile, the results showed that removing picric acid using MWCNT/CoMn_2_O_4_ was more effective than CoMn_2_O_4_ alone and confirmed the interaction effect of MWCNT nanotubes with AB_2_O_4_ nanocatalyst.

## Introduction

The rapid technological developments, the growing world population, and the increased diversification of industries have made the issue of supplying clean water one of the most challenging issues around^1^. Water is contaminated by different substances such as antibiotics, dyes, herbicides, etc., which severely threatens the health of humans, animals, and plants^2,3^. Dyes are one of the most critical water pollutants due to their use in various industries, such as leather, paper, and food^4,5^. Picric acid, or 2,4,6-trinitrophenol (TNP), is extensively employed in various industries such as pharmaceuticals, leather, explosives, chemicals, and dyes. Due to its high solubility in water, picric acid is one of the most significant water pollutants^6^. Due to the harmful effects of picric acid on the human body, especially the skin, kidneys, eyes, liver, and lungs, it is necessary to remove it from water^7^.

Diverse methods have been reported for removing contamination from water, such as ozonation^8^, photocatalytic treatment^9^, degradation^10,11^, adsorption/separation^12^, and the Fenton process^13^. In recent years, the use of advanced oxidation procedures to remove pollutants from the water has received much attention^14–16^. Pollutants are removed using the progressive oxidation method by converting them into active radicals, such as sulfate (SO_4_^·−^) and hydroxyl radicals (^·^OH). The activation of persulfate and peroxymonosulfate (PMS), as well as covering peroxodisulfate (PDS), is one of the most effective sources for the production of SO_4_^·−^ radical^17^.

However, we used PMS to degrade picric acid due to its availability, high activity, and cheapness. In recent decades, the spinel oxide AB_2_O_4_ (A/B = transition metal ions) catalysts and their composites have been utilized extensively because of their excellent synergistic effect between two kinds of metallic ions for removing water pollutants^18,19^. Using carbon nanotubes, due to their unique properties, such as high thermal conductivity and mechanical strength, as a substrate for the stabilization and stability of nanoparticles has received much attention from researchers^20,21^. Many reports have been presented in the field fabrication of CoMn_2_O_4_ nanoparticles based on various carbon materials, including pg-C_3_N_4_/CoMn_2_O_4_^22^, CoMn_2_O_4_@N-rGA^23^, CoMn_2_O_4_/NC^24^, and CoMn_2_O_4_/C hollow spheres^25^. Our findings show that no research has been reported on utilizing MWCNT/CoMn_2_O_4_ nanostructure in the removal reaction of picric acid through activation PMS. In this study, the prepared nanocomposite performed excellently in activating PMS for the degradation of picric acid as an organic contamination. The combination of cobalt and manganese nanoparticles with carbon nanotubes is due to the high activity in removing pollutants and electron transfer on carbon nanotubes. This research proposes an easy and new method for preparing of MWCNT/CoMn_2_O_4_ nanocomposite. Also, the synergistic effect CoMn_2_O_4_ of nanoparticles on multi-walled carbon nanotubes for the degradation of picric acid via the activation of peroxymonosulfate was investigated. Evaluation of picric acid degradation reaction using MWCNT/CoMn_2_O_4_ of nanocomposite in different conditions obtained good results. As far as we know, the economic preparation of nanocomposite that can activate peroxymonosulfate for the degradation of picric acid has not been presented.

## Experimental

### Chemicals

Multi-wall carbon nanotube (MWCNT), cobalt(II) chloride, manganese(II) acetate, oxalic acid, sodium hydroxide (NaOH), benzoquinone (BQ), ethanol (EtOH), potassium dichromate (K_2_Cr_2_O_7_), sodium azide (NaN_3_), and isopropyl alcohol (IPA) were procured from Sigma-Aldrich, )Germany( and Merck (Germany). Distilled water and ethanol were used as solvents in all processes. Meantime, picric acid (96% purity) was procured from Alvan Sabet Company (Iran).

### Instrumentation

Infrared spectra were obtained utilizing a (Shimadzu IR-640 spectrometer, Japan). XRD diffraction patterns were identified with a (Philips-PW 1730, Germany). Field Emission Scanning Electron Microscope (FE-SEM) and sample mapping were prepared using a (TESCAN MIRA3 microscope, Czech Republic). Transmission electron microscopy (TEM) was recorded using (Philips EM 208S, Netherlands). The images of the surface roughness of the nanostructure were obtained using the Atomic Force Microscope (AFM, Brisk, Germany). X-ray Photoelectron Spectroscopy (XPS, EA 10 analysis system, Germany) determined the type of bonds and chemical composition. The electronic vibrations of the nanocomposite and the degradation efficiency were investigated with (Labmann LMSP UV-1200, Canada).

### Fabrication of CoMn_2_O_4_ nanoparticles

The CoMn_2_O_4_ nanoparticles were fabricated in conformity with Wang et al.'s report with a few corrections^22^. To form a pure solution, 1.74 g of Mn(CH_3_CO_2_)_2_ and 650 mg of CoCl_2_ were dissolved in a mixture of water–ethanol with a ratio (1:1). Then, 1 g of oxalic acid was added to the medium reaction under vigorous stirring. After a while, to adjust the pH of the solution to 7 that 1 M of sodium hydroxide was used slowly dropwise, and the resulting solution was stirred at 80 °C for 10 h. After cooling down naturally, the black color as prepared was filtered, rinsed many times with ethanol/water, and dried at 80 °C. Eventually, the compound prepared was calcined for 3 h at 500 °C.

### Fabrication of MWCNT/CoMn_2_O_4_ nanocomposite

**Figure 1:**
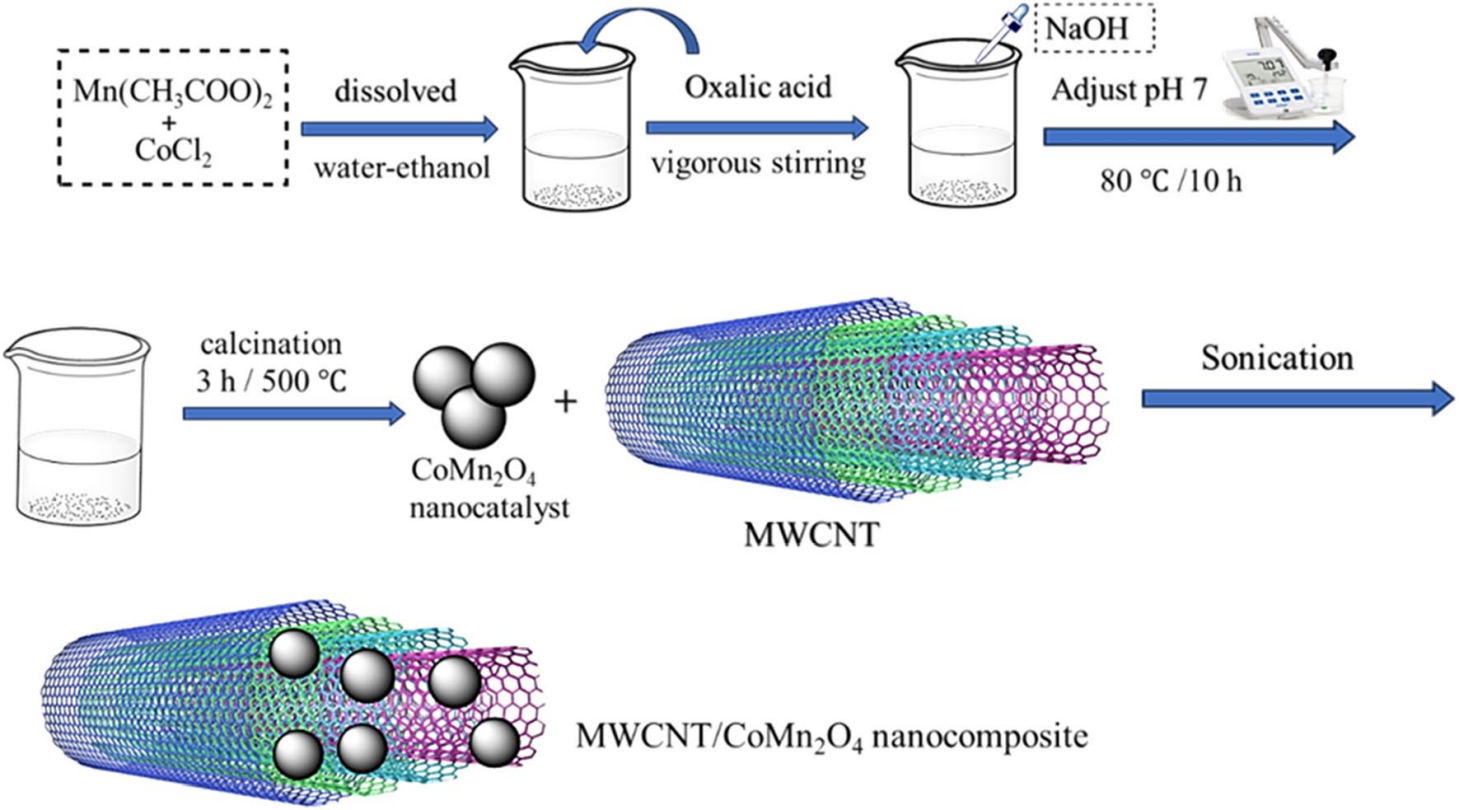
Synthetic approach for the synthesis of MWCNT/CoMn_2_O_4_ nanocomposite.

In the fabrication of this nanocomposite, 20 mg of MWCNT was added in 10 mL of ethanol to prepare a suspension solution via sonication for 60 min (Fig. 1). Then, 20 mg of as-obtained CoMn_2_O_4_ was added to the reaction mixture and sonicated (30 min) to significant adsorption of CoMn_2_O_4_ nanoparticles on the multi-wall carbon nanotubes. Next, the process admixture was often filtered and rinsed with ethanol/water. Eventually, it was dried at 80 °C for 12 h to produce MWCNT/CoMn_2_O_4_ nanocomposite^26^.

### Degradation test

The degradation process of picric acid was investigated in the presence of various dosages of MWCNT/CoMn_2_O_4_ nanocomposite as a PMS activator. The removal reaction of picric acid was checked under different conditions and using various nanocomposite dosages as a PMS activator. In the optimum condition, the removal reaction of picric acid by adding 0.05 g/L MWCNT/CoMn_2_O_4_ was put into 100 mL of picric acid solution (10 mg/L) in 30 min until it reached the adsorption–desorption equilibrium. After a while, to generate reactive species oxygen (ROS), PMS (0.3 g/L) was added to the primal process admixture. In the end, the degradation reaction was investigated via a UV–Vis spectrophotometer. The prominent absorbance peak of picric acid was observed at 354 nm, and due to the following relation, the removal efficiency was calculated:

$$\text{Removal efficiency }\left(\%\right){=}\frac{{\text{C}}{\text{t}} - {\text{C}}{\text{o}}}{{\text{C}}{\text{o}}} \times {100}$$

C_o_ and C_t_ are the final and initial picric acid concentrations, respectively.

## Results and discussion

In addition to the scientific findings in developing nanoparticles to degrade organic pollutants^27–31^, in the present research, a new strategy for the preparation of MWCNT/CoMn_2_O_4_ nanocomposite for the degradation of picric acid through the activation of peroxymonosulfate has been presented. The strategy of MWCNT/CoMn_2_O_4_ of nanocomposite synthesis consists of 2 steps, in which CoMn_2_O_4_ nanoparticles were prepared in step 1. In step 2, for the synthesis of MWCNT/CoMn_2_O_4_ nanocomposite, CoMn_2_O_4_ nanoparticles prepared using ultrasonic waves were placed on the walls of carbon nanotubes. The unique properties of carbon nanotubes have caused changes in the performance of nanoparticles and increased catalytic power. Therefore, the performance of the synthesized nanocomposite was evaluated in the process of picric acid degradation as an organic pollutant through peroxymonosulfate activation. In comparing this nanocomposite with other nanocomposites^32–36^, we can point out its distinctive features such as easy preparation, short degradation time, structural checking via various analyses, especially XPS and AFM spectrum, high degradation efficiency, excellent performance by few dosages of the nanocomposite in the degradation process, degradation with the easy method without the need to sunlight and radiation, and investigating the removal picric acid as a vital contamination in drug and industries.

### Characterization

**Figure 2:**
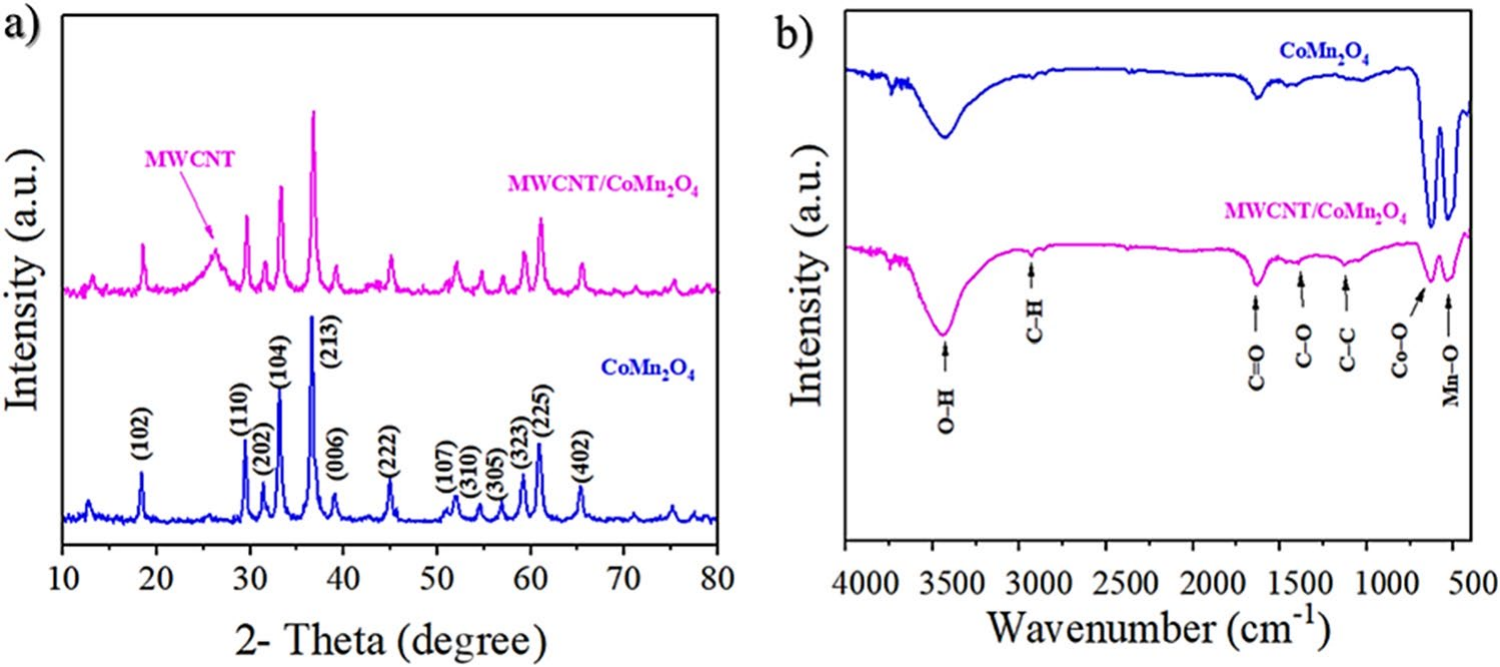
XRD (**a**) and FT-IR (**b**) pattern of CoMn_2_O_4_ and MWCNT/CoMn_2_O_4_ nanostructures.

The X-ray diffraction pattern has been used to identify and distinguish the crystal structure and crystal size of the synthesized CoMn_2_O_4_ nanocatalyst and MWCNT/CoMn_2_O_4_ nanocomposite. As shown in Fig. 2b, the different peaks emerged in 18.67$^\circ$, 29.37$^\circ$, 31.57$^\circ$, 33.32$^\circ$, 44.82$^\circ$, 61.12$^\circ$, and 65.53$^\circ$ were related to (102), (110), (202), (213), (222), (225), and (402) in CoMn_2_O_4_ nanoparticles surfaces (JCPDS No. 29-1487), respectively. Among various peaks, 36.72$^\circ$(104) was obtained, corresponding to the CoMn_2_O_4_ nanocatalyst^37^. In addition, a peak at 26.78$^\circ$ can belong to multi-wall nanocarbon nanotubes^38^. Based on the findings from XRD patterns, both CoMn_2_O_4_ and MWCNT/CoMn_2_O_4_ nanocomposites were successfully prepared.

The FT-IR spectrum was investigated to find the functional groups in CoMn_2_O_4_ and MWCNT/CoMn_2_O_4_ nanocomposite from 400 to 4000 cm^−1^. As indicated in Fig. 2a, a strong peak near 3445 cm^−1^ corresponding to O–H stretching appeared due to the absorption of H_2_O on the nanocomposite surface. However, there are various peaks at 1126, 1390, 1627, and 2930 cm^−1^, which were related to C–C, C–O, C=O, and C–H, respectively. MWCNT/CoMn_2_O_4_ nanocomposite has two peaks at 626 and 543 cm^−1^ because of Co–O and Mn–O bonds, respectively^39,40^. Based on the peaks in the nanoparticle and nanocomposite structure (related to each of the functional groups) that are characteristic of the pure and correct form of the products after synthesis, it may be concluded that the compounds were produced adequately.

**Figure 3:**
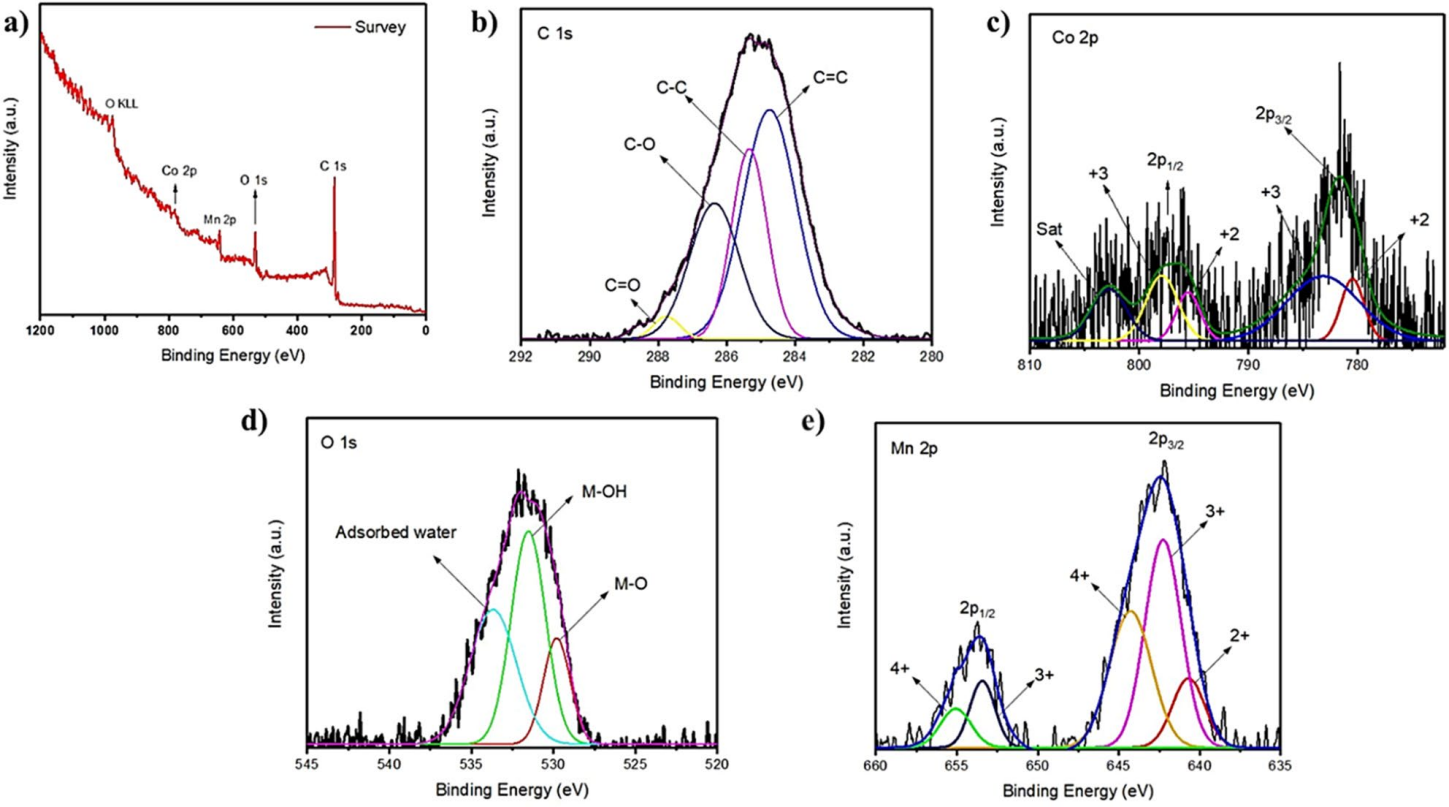
XPS spectra of the MWCNT/CoMn_2_O_4_ nanostructure: survey scan (**a**), C 1s (**b**), Co 2p (**c**), O 1s (**d**), and Mn 2p (**e**).

The electronic states and elemental composition of the CoMn_2_O_4_ and MWCNT/CoMn_2_O_4_ nanostructure were assessed by XPS, as shown in Fig. 3. The full scan XPS spectrum of the nanostructure (Fig. 3a) displayed the existence of elements C, Co, Mn, and O in the CoMn_2_O_4_ and MWCNT/CoMn_2_O_4_ nanostructure. The high-resolution spectrum of C 1s is divided into four peaks (Fig. 3b), which involving energy bands of 291.40 eV are related to the 284.7 eV to the C=C bond 285.3 eV to the C−C bond, 286.3 eV to the C–O bond, and 287.7 eV to the C=O bond. Figure 3c indicates the high-resolution XPS spectrum of Co 2p. The Co atom was demonstrated in two oxidation states, Co^+2^ and Co^+3^, in the structure of the MWCNT/CoMn_2_O_4_ compound. Two peaks correspond to Co 2p1/2 and Co 2p3/2. The peak at 932.15 eV is assigned to Co^2+^, while two peaks at 936.4 and 954.17 eV can attributed to Co^3+^. Peaks at 529.7, 531.5, and 533.6 eV in the O 1s XPS spectra (Fig. 3d) can be linked to hydroxyl groups, metal–oxygen bonds, and absorbed water, respectively. High-resolution XPS spectrum of Mn appeared in Mn^2+^, Mn^3+^, and Mn^4+^ oxidation states (Fig. 3e). The peak related to Mn^2+^ appeared at 640.6, while the two peaks linked to Mn^3+^ were revealed at 642.2 and 653.4 eV. In addition, peaks belonging to Mn^4+^ were observed at 644.2 and 655 eV.

**Figure 4:**
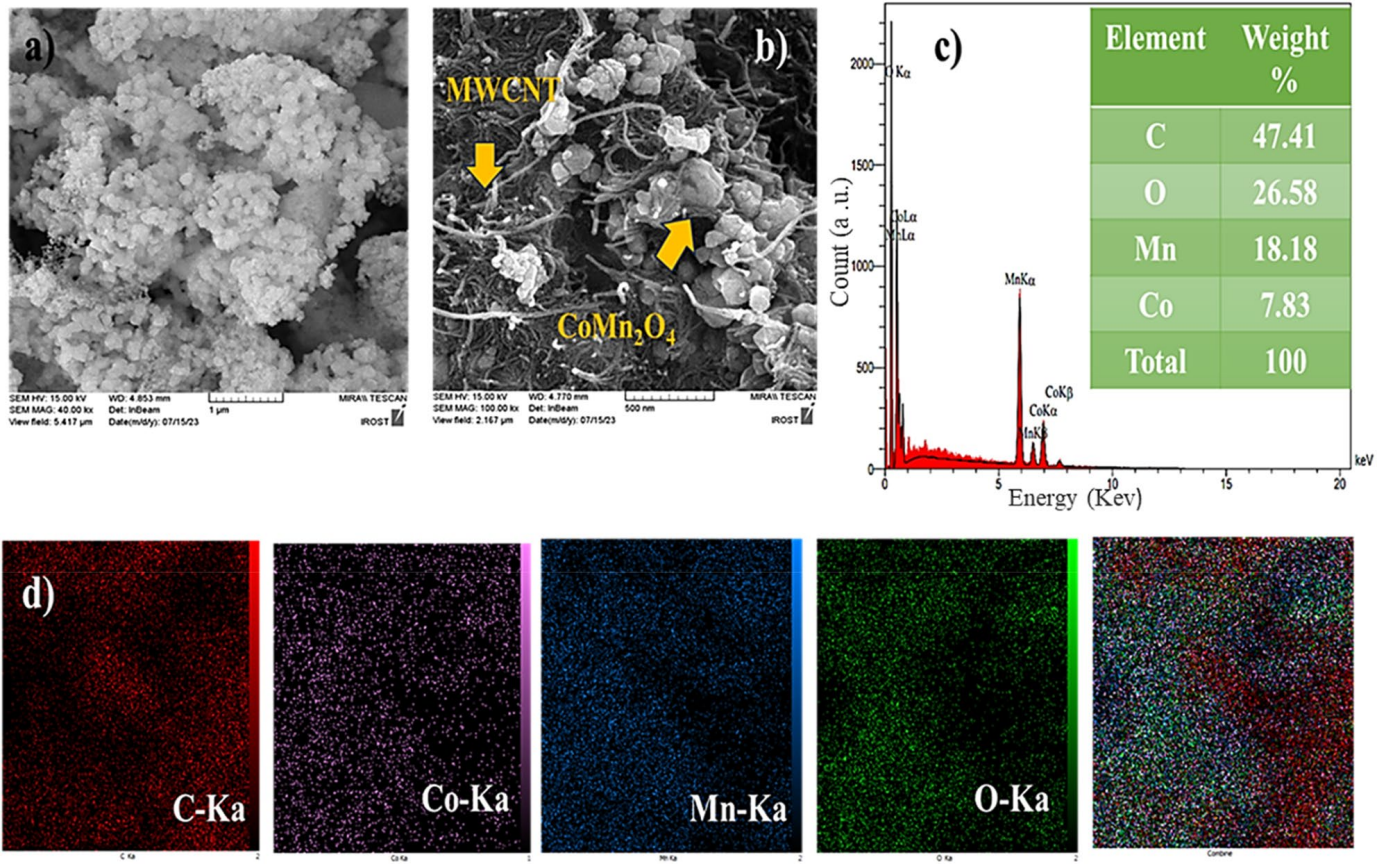
FESEM images CoMn_2_O_4_ (**a**), MWCNT/ CoMn_2_O_4_ (**b**), EDX (**c**), and SEM-mapping (**d**) of the nanocomposite.

FESEM, TEM, and AFM investigated the structure and surface morphology of CoMn_2_O_4_ and MWCNT/CoMn_2_O_4_ nanocomposite. As displayed in Fig. 4a, the CoMn_2_O_4_ nanoparticles have a symmetrical geometry morphology with cuboid microcrystals. Also, the thickness of nanoparticles was evaluated between 0.1 and 1 µm. Meanwhile, the MWCNT appeared in tubular morphology, corroborating the loading of CoMn_2_O_4_ nanostructures on the MWCNT (Fig. 4b). Elemental distribution maps and EDX spectra were utilized to measure the elements′ distribution, and nanocomposite purity. Both analyses' results acknowledged the high purity of carbon, cobalt, manganese, and oxygen elements in the nanocomposite structure (Fig. 4c). The result obtained from elemental mapping analysis is exhibited in Fig. 4d.

**Figure 5:**
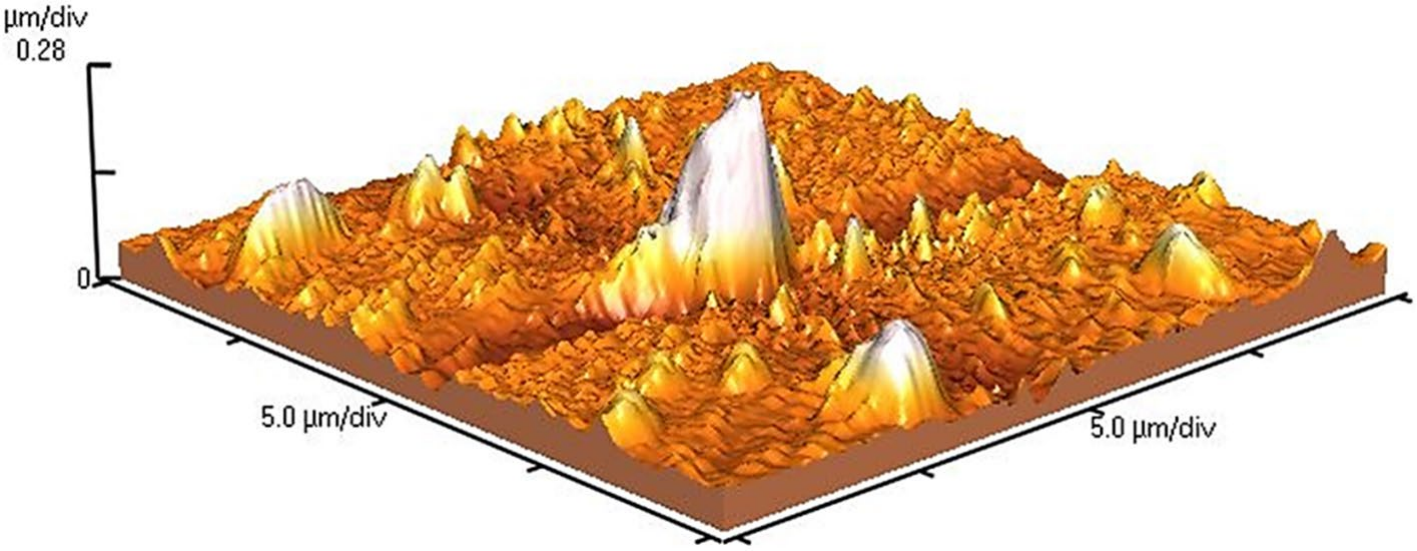
AFM image of MWCNT/CoMn_2_O_4_.

An atomic force microscope (AFM) is an essential and helpful instrument for checking the topography of the surface on a nanometer scale. This microscope is used to image the roughness of the nanostructure surface and provide two- and three-dimensional images. The results obtained from the AFM analysis of the nanocomposite structure are indicated in Fig. 5. As shown in Fig. 5, the CoMn_2_O_4_ nanoparticles have mountain-like morphology with white edges. In contrast, MWCNT has a non-intersecting surface on which CoMn_2_O_4_ nanoparticles were placed. In the meantime, the surface roughness of the MWCNT/COMn_2_O_4_ nanocomposite was estimated at 25.62 nm.

**Figure 6:**
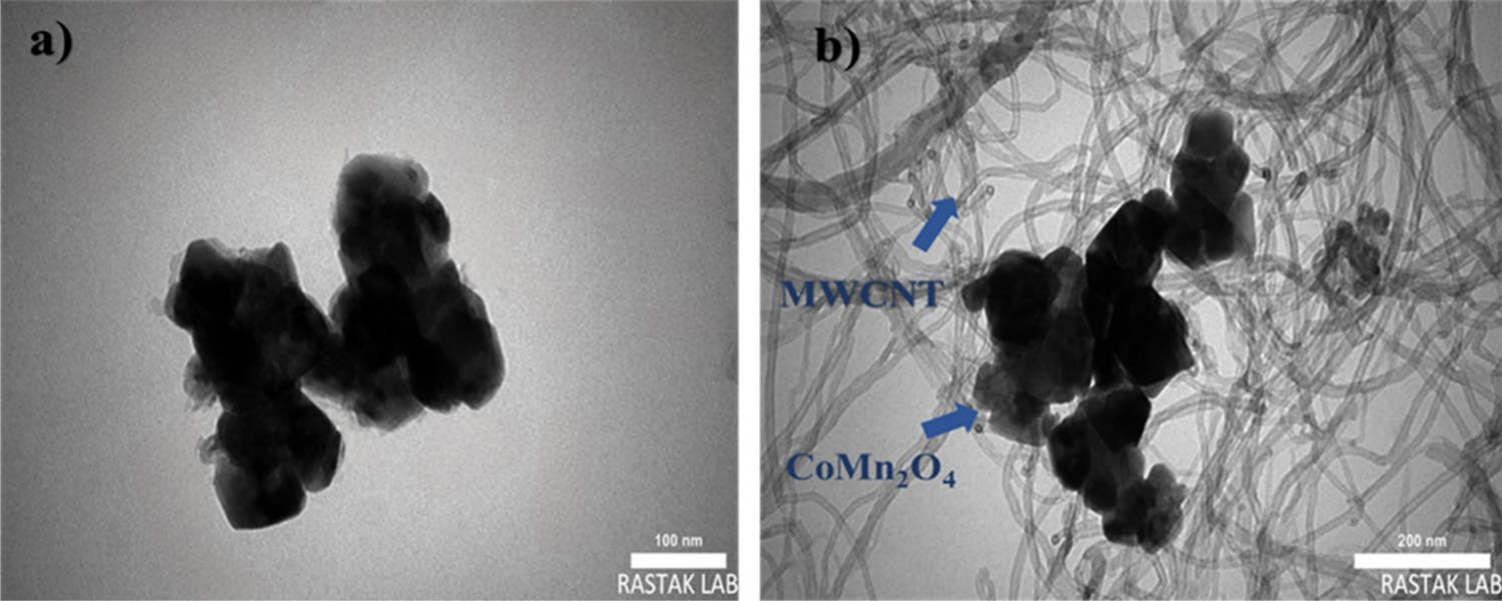
TEM images of CoMn_2_O_4_ (**a**) and MWCNT/CoMn_2_O_4_ nanostructures (**b**).

TEM is applied to clarify the structure and the morphology of MWCNT/CoMn_2_O_4_ nanocomposite even more. The results of the images can be seen in the Fig. 6. These images depict nanoparticles correctly placed on multi-wall carbon nanotubes with high magnification. CoMn_2_O_4_ nanoparticles are loaded in black aggregates similar to those between the walls of carbon nanotubes. Compared to SEM, the TEM analysis gives a detailed look at the nanocomposite.

### Degradation efficiency

**Figure 7:**
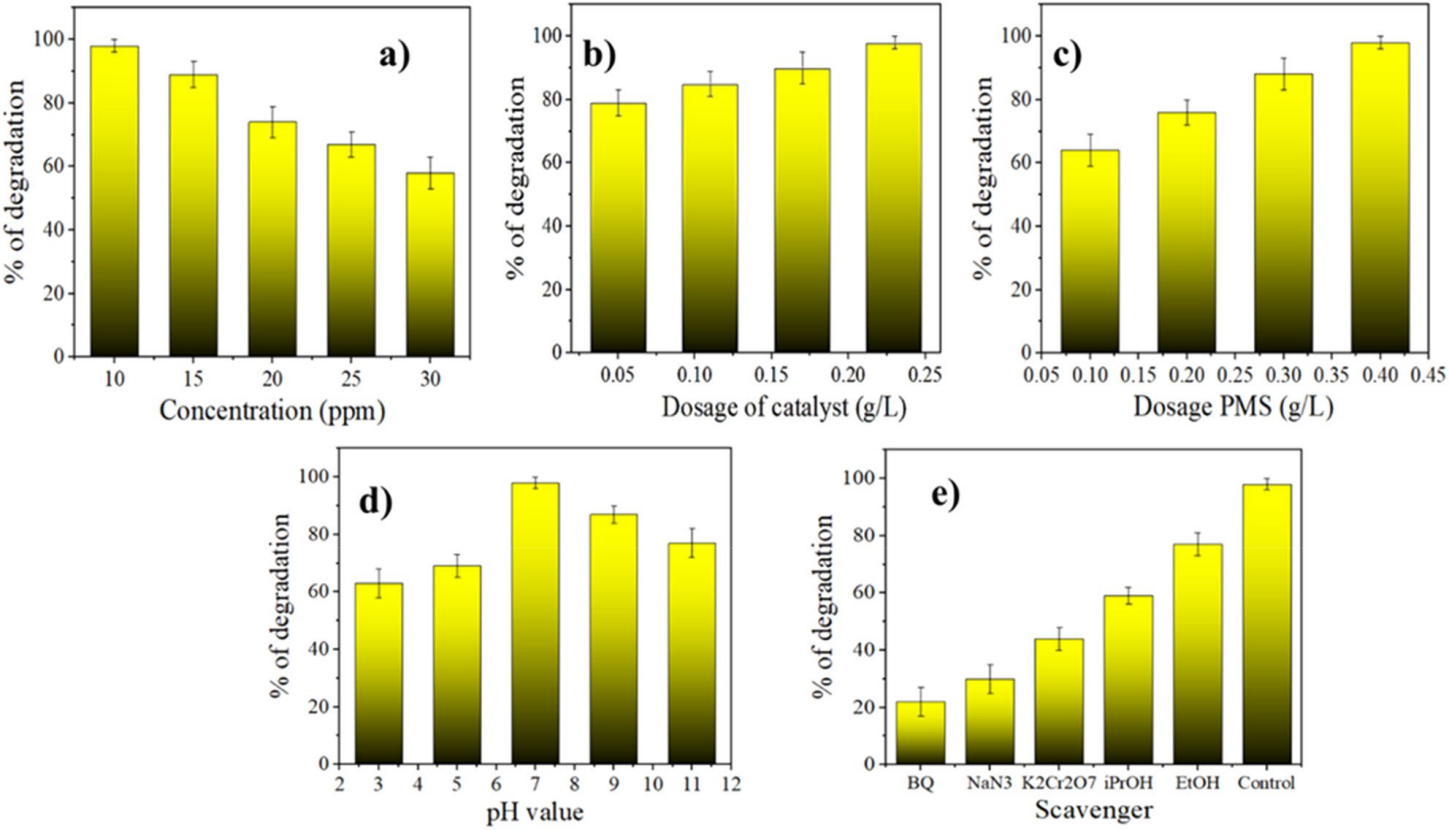
Impact of various factories in % of remove: the concentration of CV (**a**), a dosage of catalyst (**b**), the dosage of PMS (**c**), pH (**d**), and scavenger (**e**).

Due to the severe water scarcity in many places, the destruction of organic pollutants, particularly picric acid, which is detrimental to the human body, animals, and plants, has come to notice. Hence, MWCNT/CoMn_2_O_4_ nanocomposite, which is totally synthesized and characterized afterward, is employed in this study to act as the PMS activator to degrade picric acid efficiently. The efficiency of the nanocomposite in the production of picric acid hydrolysis was determined by performing the test in the presence of various factors, and the results are shown in Fig. 7. The consequences displayed that utilization of the CoMn_2_O_4_ caused degradation of picric acid with yield degradation of 24% within 120 min. Without PMS, MWCNT/CoMn_2_O_4_ nanocomposite can only remove about 12% of picric acid in 80 min. The reaction of the picric acid degradation using both PMS (0.3 g/L) and MWCNT/CoMn_2_O_4_ nanocomposite (0.05 g/L) went well as expected. Also, the degradation of the picric acid takes place within 47 min. In the degradation of picric acid from wastewater, the offered nanocomposite can compete with the other nanocomposites (Table 1).

**Table 1:** The studies of picric acid removal with the utilization of different nanocomposites.

Nanocomposites	Treatment	Efficiency (%)	Process conditions	References
rGO-TiO_2_	H_2_O_2_	100	[C_PA_] = 200 ppm, [cat] = 50 mg/L, [H_2_O_2_] = 50 mg/L, 15 min	^37^
Nano-CeO_2_-loaded chitosan-bocglycine zinc complex	H_2_O_2_	100	[C_PA_] = 200 ppm, [cat] = 50 mg/L, [H_2_O_2_] = 20 mM/L, 35 min	^38^
Pd@TiO_2_	Catalyst	100	[C_PA_] = 500 ppm, [cat] = 20 mg/L, 70 min	^39^
Ag/ZnO	Photocatalyst	90	[C_PA_] = 10 ppm, [cat] = 60 mg/L, 150 min	^40^
MWCNT-Chi	Catalyst	90	[C_PA_] = 100 ppm, [cat] = 20 mg/L, 240 min	^41^
AV-TiO_2_	Photocatalyst	97	[C_PA_] = 500 ppm, [cat] = 20 mg/L, 120 min	^42^
**MWCNT/CoMn**_**2**_**O**_**4**_	**PMS**	**98**	**[C**_**PA**_**] = 10 ppm, [cat] = 0.05 g/L, [PMS] = 0.3 g/L, 47 min**	**Current work**

Significant values in bold.

### Impact of various factors

The effect of picric acid concentration was investigated from 10 to 30 ppm in the picric acid degradation, and the degradation yield of picric acid reduced from 98 to 58% (Fig. 7a). This can be related to the saturation of reactive levels in the nanocomposite, which reduces ROS. Also, the effect of the MWCNT/CoMn_2_O_4_ nanocomposite dosage was measured in dosages of 0.05, 0.1, 0.15, and 0.2 g/L in the picric acid removal. As indicated in Fig. 7b, because of efficient nanoparticles of cobalt, manganese and the high electrical conductivity of carbon nanotubes on the level of the nanocomposite, the picric acid removal increased between 79 and 98% within 47 min. Nonetheless, the degradation process of picric acid was evaluated at PMS concentrations of 0.1, 0.2, 0.3, and 0.4 g/L, and picric acid removal was enhanced from 64 to 98% (Fig. 7c). The good impact of this factor is because of the delivery of reactive oxygen species, including HO^·^, O_2_^·−^, ^1^O_2_, and SO_4_^·−^. The degradation reactions of picric acid using MWCNT/CoMn_2_O_4_ nanostructure at different pHs from 3 to 10 were studied and displayed in Fig. 7d. The findings acquired from the impact of pH on the removal reactions specified that the removal of picric acid occurred more successfully in alkaline than in acidic pH. After that, the scavenger experiment was utilized to investigate the radical species included in the removal reaction. We conducted studies on the removal reaction of picric acid by use of various scavengers such as sodium azide (NaN_3_), ethanol, benzoquinone (BQ), isopropyl alcohol (IPA), and potassium dichromate (K_2_Cr_2_O_7_). According to Fig. 7e, the species HO^·^ and SO_4_^·−^ are mostly noticed as the significant radicals in degradation reactions, and the catalytic performance must be a low weaken using of EtOH for scavenging HO^·^ and SO_4_^·−^. As provided in Fig. 7e, when EtOH was used as a scavenger, the picric acid degraded by efficacy 77% after 47 min. In conjunction with it, the yield removal of picric acid using IPA was 59%. In conformity with the finding, it was determined that EtOH and IPA have an excellent scavenging action compared to sulfate and hydroxyl species. To check the activity of nanostructure for practical applications in the removal reaction, we performed the process on a large scale. For this reason, we utilized picric acid (10 mg/L) of MWCNT/CoMn_2_O_4_ nanostructure (0.05 g/L) and PMS (0.3 g/L) to carry out the removal reaction on a larger scale, which caused a more effective remove of picric acid in 47 min. The outcomes indicated that this process is practically stable and manageable.

### Proffered mechanism of picric acid removal

**Figure 8:**
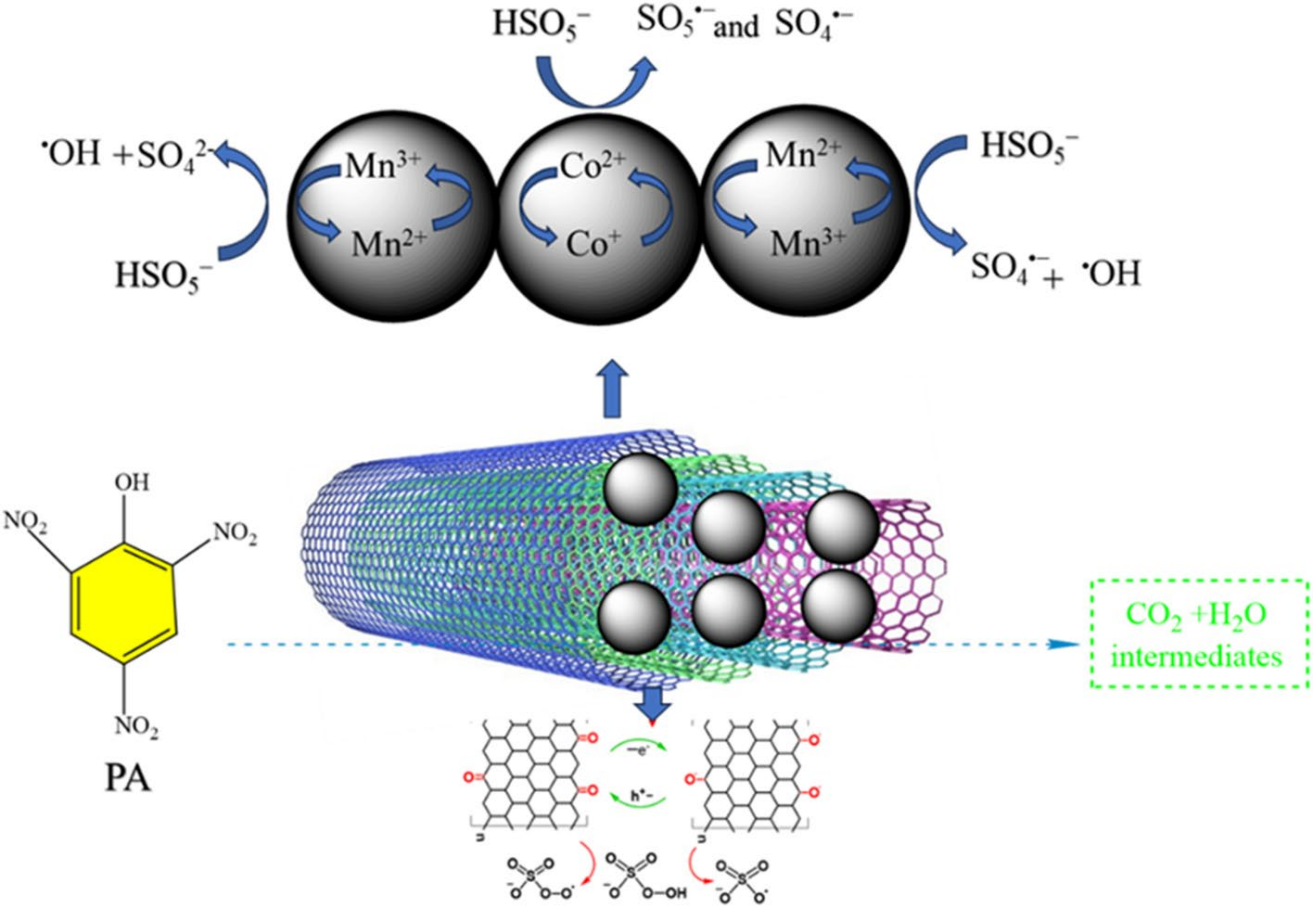
The mechanism for PA removal was presented using the MWCNT/CoMn_2_O_4_ through the activation of PMS.

Based on the findings, a possible mechanism was presented for the degradation of picric acid via radicals and electron transforms (Fig. 8). The results indicated that placing CoMn_2_O_4_ nanoparticles onto the multi-wall carbon nanotubes caused an increase in transform electron and degradation yield. Meanwhile, there are many methods for producing free radicals, including ozone, hydrogen peroxide, and persulfate^41^. In this study, MWCNT/CoMn_2_O_4_ nanocomposite removed the organic pollutant by using PMS to produce ^·^OH, SO_4_^·−^, ^1^O_2_, and O_2_^·−^ prepared radicals. However, the SO_4_^·−^ radical reacts highly with organic contaminations because of its excellent oxidative potential^42^. Also, the scavenging test was utilized to show the roles of ROS in the degradation reaction. In the removal process, different scavengers, such as sodium azide (NaN_3_), potassium dichromate (K_2_Cr_2_O_7_), isopropyl alcohol (^i^PrOH), ethanol (EtOH), and benzoquinone (BQ), have been checked to trap radicals. According to the findings, the degradation process happened with a higher yield in ethanol than in the presence of other scavengers. The prominent radicals in the reaction are OH^·^ and SO_4_^·−^ due to the ethanol being a trapper of OH^·^ and SO_4_^·−^. The manganese and cobalt on the surface of MWCNT were reacted by PMS (HSO_5_^−^) to generate SO_4_^·−^ and ^·^OH with Eqs. (1) and (2)^43^. Then, to create ^·^OH, water was reacted with SO_4_^·−^ radicals (Eq. 3). In the next step, the Co^+^ ion and SO_5_^·−^ radical were prepared for the reaction of Co^2+^ ions on the surface MWCNT/CoMn_2_O_4_ by PMS (Eq. 4). Since the Co^1+^ ion converted to Co^2+^ (Eq. 5). Meantime, Co^2+^, SO_4_^2-^, and OH^·^ were prepared from the reaction Co^1+^ ions with HSO_5_^−^ (Eq. 6)^44^. Respectively, the SO_4_^·−^ and SO_5_^·−^ were prepared from the reaction of the Mn^2+^ and Mn^3+^ ions on the surface MWCNT/CoMn_2_O_4_ with HSO_5_^−^ (Eqs. 7 and 8)^45^. However, multi-wall carbon nanotubes can have an essential obligation in electron transfer to PMS because of their distinctive properties (Eq. 9). In the end, to complete the degradation process of organic contaminant, the OH^·^ and SO_4_^·−^ radicals with the picric acid were turned into H_2_O, CO_2_, and intermediate (Eq. 10).

1 $${\text{HSO}}_{{5}}^{ - } + {\text{ e}}^{ - }\rightarrow {\text{SO}}_{{4}}^{ \cdot - } +^{ \cdot } {\text{OH}}$$

2 $${\text{HSO}}_{{5}}^{ - } + {\text{ e}}^{ - } \rightarrow{\text{SO}}_{{4}}^{{{2} - }} +^{ \cdot } {\text{OH}}$$

3 $${\text{SO}}_{{4}}^{ \cdot - } + {\text{H}}{_{{2}}{\text{O}}}\rightarrow {\text{SO}}{_{{4}}}^{{2} - } +^{ \cdot } {\text{OH }} + {\text{ H}}^{ + }$$

4 $${\text{Co}}^{{{2} + }} + {\text{ HSO}}_{{5}}^{ - } \rightarrow{\text{Co}}^{{{2} + }} + {\text{SO}}_{{5}}^{ \cdot - }$$

5 $${\text{Co}}^{{{1} + }} + {\text{HSO}}_{{5}}^{ - } \rightarrow{\text{Co}}^{{{2} + }} + {\text{ SO}}_{{4}}^{ \cdot - }$$

6 $${\text{Co}}^{{{1} + }} + {\text{HSO}}_{{5}}^{ - } \rightarrow{\text{Co}}^{{{2} + }} +^{ \cdot } {\text{OH }} + {\text{ SO}}_{{4}}^{{{2} - }}$$

7 $${\text{HSO}}_{{5}}^{ - } + {\text{Mn}}^{{{2} + }} \rightarrow{\text{SO}}_{{4}}^{ \cdot - } + {\text{ Mn}}^{{{3} + }}$$

8 $${\text{HSO}}_{{5}}^{ - } + {\text{ Mn}}^{{{3} + }} \rightarrow{\text{SO}}_{{5}}^{ \cdot - } + {\text{ Mn}}^{{{2} + }}$$

9 $${\text{HSO}}_{{5}}^{ - } + {\text{ h}}^{ + } \rightarrow{\text{SO}}_{{5}}^{ \cdot - } + {\text{ H}}^{ + }$$

10 $${\text{PA }} + {\text{ SO}}_{{4}}^{ \cdot - } /^{ \cdot } {\text{OH}} \rightarrow{\text{intermediates}} \rightarrow{\text{CO}}_{{2}} + {\text{ H}}_{{2}} {\text{O}}$$

### Reusability investigations

**Figure 9:**
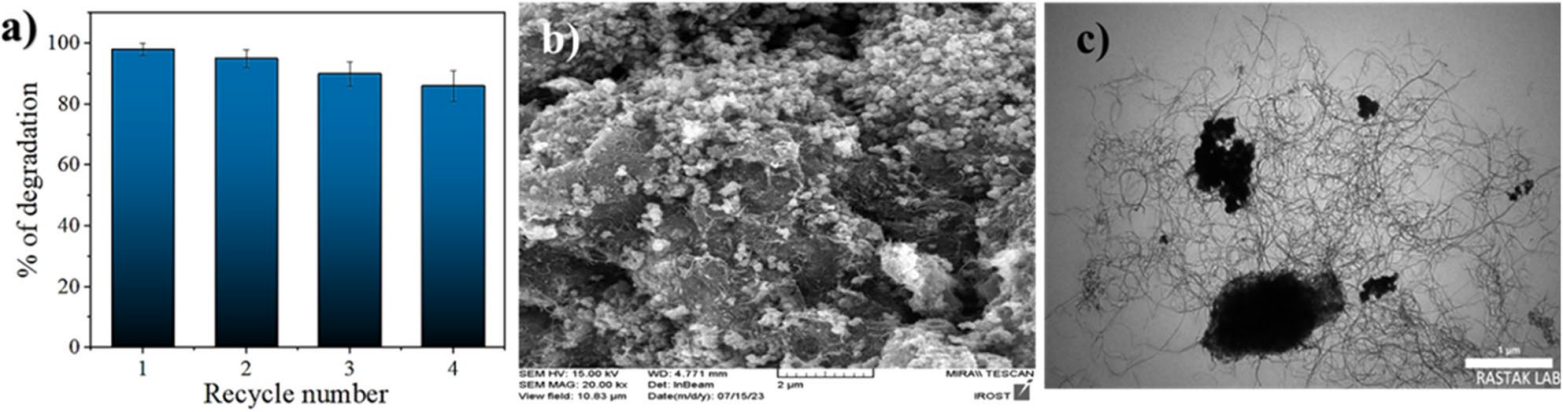
Results of recycling MWCNT/CoMn_2_O_4_ nanocomposite for PA remove reaction (**a**), FESEM (**b**), and TEM (**c**) images of the MWCNT/CoMn_2_O_4_ after 4th recycle reaction.

Compared to previous works, one of the distinguishing features of this work is the ability to reuse the nanocomposite during several cycles. In this research, to investigate the reusability of the nanocomposite after performing the desired reaction, we separated the nanocomposite via a centrifuge (2800 rpm for 15 min) from the reaction mixture, several times rinsed with water/ethanol and next dried it at 100 °C during 7 h. According to Fig. 9, the MWCNT/CoMn_2_O_4_ nanocomposite was recycled during four cycles, and no significant change in its activity was observed during the recycling process. Also, the structure of the recycled nanocomposite was studied through different analyses, such as FESEM (Fig. 9b) and TEM (Fig. 9c). Investigations showed that its structure remained almost constant.

## Conclusions

In this research, a new generation of MWCNT/CoMn_2_O_4_ nanocomposite was designed and successfully made to degrade picric acid. The results obtained through different analyzes confirmed the growth and stabilization of CoMn_2_O_4_ nanoparticles on MWCNT. The presence of cobalt and manganese active sites on nanoparticles and the growth of CoMn_2_O_4_ nanoparticles on MWCNT as a substrate showed a significant increase in catalytic activity towards the degradation of picric acid. In addition, the investigation of the reaction mechanism and various tests, especially the scavenger test, confirmed that SO_4_^·−^ and ^·^OH radicals play an essential role in the degradation of picric acid. Finally, the current study provides new insight into designing and fabricating nanoparticles on carbon nanotubes to improve water treatment.

## Data Availability

All data have been given in the article.
